# Supplementary material for: A pilot randomised trial of a brief virtual reality scenario in smokers unmotivated to quit: Assessing the feasibility of recruitment
Source: PLOS Digit Health. 2022 Jun 27;1(6):e0000060. doi: 10.1371/journal.pdig.0000060 (PMC9931367; doi:10.1371/journal.pdig.0000060)
Supplement: S1 Table — (DOCX) [file pdig.0000060.s003.docx]

**S1 Table**

*Table A.* Sensitivity analysis with the outcome variables operationalised as continuous (as opposed to categorical) variables.

|  | **Intervention (n=30)** | **Control (n=30)** | ***p*-value*** |
| --- | --- | --- | --- |
| The scenario made me feel angry (1-7), mean (*SD*) | 2.10 (*1.79*) | 1.87 (*1.20*) | 0.56 |
| The scenario made me feel distressed (1-7), mean (*SD*) | 2.63 (*1.90*) | 2.53 (*1.83*) | 0.84 |
| The scenario was useful to me (1-7), mean (*SD*) | 4.57 (*1.45*) | 4.53 (*1.20*) | 0.92 |
| Perceived susceptibility to cancer (1-5), mean (*SD*) | 4.27 (*0.69*) | 4.20 (*0.81*) | 0.73 |
| Perceived susceptibility to heart disease (1-5), mean (*SD*) | 3.83 (*0.87*) | 4.03 (*0.81*) | 0.36 |
| Perceived susceptibility to lung disease (1-5), mean (*SD*) | 4.30 (*0.70*) | 4.40 (*0.86*) | 0.62 |
| Cancer response-efficacy (1-7), mean (*SD*) | 6.07 (*1.26*) | 5.93 (*1.01*) | 0.65 |
| Heart disease response-efficacy (1-7), mean (*SD*) | 5.40 (*1.69*) | 5.47 (*1.57*) | 0.88 |
| Lung disease response-efficacy (1-7), mean (*SD*) | 6.33 (*1.03*) | 6.17 (*1.02*) | 0.53 |
| Quitting self-efficacy (1-5), mean (*SD*) | 2.30 (*0.95*) | 3.03 (*1.07*) | 0.006** |

*Note.* * Linear regression analyses. ** P-vales significant at the 0.05 level (two-tailed). SD = Standard deviation.

*Table B.* Bivariate correlations between the acceptability and susceptibility indicators.

|  | Perceived susceptibility to cancer | Perceived susceptibility to heart disease | Perceived susceptibility to lung disease |
| --- | --- | --- | --- |
| The scenario made me feel angry | .09 | .05 | .03 |
| The scenario made me feel distressed | .13 | .15 | .11 |
| The scenario was useful to me | -.08 | -.07 | -.04 |

*Note.* * P-values significant at the 0.05 level (two-tailed). Please note that none of the p-values were significant.
