# Supplementary material for: A pilot randomised trial of a brief virtual reality scenario in smokers unmotivated to quit: Assessing the feasibility of recruitment
Source: PLOS Digit Health. 2022 Jun 27;1(6):e0000060. doi: 10.1371/journal.pdig.0000060 (PMC9931367; doi:10.1371/journal.pdig.0000060)
Supplement: Attachment — Submitted filename: Responses to reviewer comments 21.03.22.docx [file pdig.0000060.s004.docx]

**Editor**

The reviewers see potential in your submission, however, they also highlighted a number of issues including:

- a lack of detial in describing immersive VR and the intervention, including behaviour change techniques used

- the decision to dichotomise variables in the analysis

- the discussion being short and not referring at all to research question 3.

We thank the editor for the helpful feedback and for the opportunity to revise our paper. We now provide more detail on immersive VR (both in general and in relation to our VR scenario; p.3-4) and the content of our VR scenario (including the behaviour change techniques; p.3-4 and p.8).

“Although feelings of immersion (e.g., transportation to a different, virtual environment, losing track of time) are characteristic of VR more broadly, the level of immersion experienced by users differs depending on the specific technology used, the number of senses engaged (e.g., vision, hearing, smell) and the level of interactivity (e.g., whether users can interact with objects in the virtual environment through, for example, a joystick) (Miller and Bugnariu, 2016).”

“We therefore helped develop an immersive, brief VR scenario – suited for delivery in smokers’ own homes and accessible via commercially available head-mounted displays without involving a joystick – grounded in the Extended Parallel Process Model (EPPM) (Witte, 1992)., The EPPM proposes that the effect of threatening health messages (e.g., information or communications highlighting that smoking causes lung cancer) on people’s motivation to protect themselves against said threat (e.g., through behaviour change) depends on two things: 1) people’s emotional responses to the message, and 2) their response- and self-efficacy beliefs (i.e., beliefs that the suggested action to avoid the threat is effective and that one has the ability to act). Previous research guided by the EPPM involving pictorial health warning labels on cigarette packages has found an association of greater self-efficacy and stronger emotional responses to health warning labels with prospective quit attempts (Thrasher et al., 2016). We therefore developed a hospital-based VR scenario, in which participants are invited to attend a consultation with a chest physician in a hospital clinic room and are told that they have had an abnormal chest scan which requires follow-up (i.e., a threatening health message). They are subsequently presented with a brief, written message to boost their response- and quitting self-efficacy. The hypothesised mechanism of action of the EPPM-based scenario is increased susceptibility to smoking-related diseases (i.e., a health threat) combined with increased response- and quitting self-efficacy, which are jointly expected to increase participants’ intention to stop smoking. With regards to the level of immersion, users are not able to interact with the physician via, for example, a joystick and the primary senses engaged are vision and hearing (but not smell, taste or haptics).”

“See the Supplementary Materials 2 for the behaviour change techniques (BCTs) included in the intervention scenario, coded against a 44-item taxonomy of BCTs used in behavioural smoking cessation interventions (Michie et al., 2011).”

“Due to the potential of VR to evoke strong sensory and emotional experiences – e.g., prior research shows it can reliably induce smoking urges (Pericot-Valverde et al., 2016) – we expected the immersive VR scenario to evoke strong emotional responses to the health message. Although we did not include a 2D video control, we expected emotional responses to be stronger than if, for example, viewing a 2D video with the same health message.”

We now present the results from a series of sensitivity analyses in the Supplementary Materials 3, in which we do not dichotomise the ordinal variables (referred to on p.10 and p.13):

“As a result of the peer review process, we also present the results without dichotomisation in the Supplementary Materials 3.”

“The results remained similar in the sensitivity analysis without dichotomisation (see the Supplementary Materials 3).”

We now provide a lengthier discussion of the results (and measure) pertaining to the third research question on p.13:

“The perceived susceptibility and response-efficacy to cancer, heart disease, and lung disease were comparably high across arms, which may be indicative of a ceiling effect (i.e., participants already felt moderately to highly susceptible). Quitting self-efficacy and intention to stop smoking were comparably low across arms. There are different plausible interpretations of this finding. First, the brief VR scenario may have been insufficiently immersive or persuasive for boosting smokers’ self-efficacy and therefore also their motivation to stop. Second, smokers may have slightly rushed through the post-task survey and hence missed the link with more information about smoking cessation. This merits further exploration in, for example, a study using think aloud methodology, with smokers verbalising their thoughts and impressions whilst viewing the VR scenario.”

**Journal requirements**

1. Please amend your detailed Financial Disclosure statement. This is published with the article, therefore should be completed in full sentences and contain the exact wording you wish to be published.

State what role the funders took in the study. If the funders had no role in your study, please state: “The funders had no role in study design, data collection and analysis, decision to publish, or preparation of the manuscript.”

2. Please update the completed 'Competing Interests' statement. Please declare all competing interests beginning with the statement "I have read the journal's policy and the authors of this manuscript have the following competing interests:"

3. Please provide separate figure files in .tif or .eps format only and remove any figures embedded in your manuscript file. Please ensure that all files are under our size limit of 20MB.

We have included updated Financial Disclosure and Competing Interests statements in the cover letter.

“OP, DK, and TJ receive salary support from Cancer Research UK (C1417/A22962). JB, OP, DK and TJ are members of SPECTRUM – a UK Prevention Research Partnership Consortium (MR/S037519/1).”

“OP, DK, and TJ have no conflicts of interest to declare. JB has received unrestricted research funding to study smoking cessation from manufacturers of smoking cessation medications (Pfizer and Johnson & Johnson). All authors declare no financial links with tobacco companies or e‐cigarette manufacturers or their representatives.”

We have removed the figures from the manuscript file and instead uploaded these separately in .tif format.

**Reviewer #1**

Thanks for the opportunity to review this interesting manuscript about a novel VR-based strategy to reach and help smokers unmotivated to quit. The manuscript is easy to read and generally offers a sound overview of the research that was conducted. I would advise the authors to take a look at the feedback points below, to make further improvements to their manuscript.

We thank the reviewer for their helpful feedback on our paper, which has helped strengthen it.

- the introduction is well-written and offers a clear overview of the main concepts and rationale for main choices. At several points, the introduction raises questions, but these are always answered when one continues reading the next sections. So, overall the introduction is on point and well-substantiated with references.

Thank you.

- the methods section includes a rather detailed account of how the pilot trial was set up, how inclusion and recruitment took place, and what data was collected in both study arms. Yet, the section describing the intervention conditions could elaborate more on the specific working mechanism of the immersive VR component (e.g. in comparison to watching a video of a physician talking to you); what makes VR potentially more effective than other kind of media outlets/channels?

We have now added the following in the Methods on p.8:

“Due to the potential of VR to evoke strong sensory and emotional experiences – e.g., prior research shows it can reliably induce smoking urges (Pericot-Valverde et al., 2016) – we expected the immersive VR scenario to evoke strong emotional responses to the health message. Although we did not include a 2D video control, we expected emotional responses to be stronger than if, for example, viewing a 2D video with the same health message.”

- although the results and conclusions seem clear, it would be interesting to read more about potential improvements to the active VR scenario. According to the authors, what may be the most promising application of VR for smoking cessation, and which target population should this be particularly aimed at?

Thank you – we have added a suggested area of improvement on p.15. As this pilot study doesn’t directly address different applications of VR for smoking cessation across different target populations, we politely disagree with the suggestion to add suggestions in relation to this (as they wouldn’t follow directly from our results).

“Areas of improvement may, for example, include the addition of functionality to allow users to directly interact with the physician (e.g., through selecting different options via a joystick)”.

Minor comments

- there is some redundancy in the information provided in the second paragraph of section 2.4 measures and procedures.

This paragraph has now been shortened.

- the part describing and justifying the recruitment goal is somewhat confusing: could you make it more explicit how the 60 participants in 3 months goal resulted from the full-trial calculations provided?

We have now added the following on p.10:

”Therefore, based on the above assumptions, being able to recruit 60 participants within three months from the trial start date (or ~200 participants with added resource), this would ensure we would be able to recruit ~1600 participants within 24 months from the trial start date.”

- results: could you clarify why only 103 out of 376 eligible participants were randomized?

This has now been clarified on p.10. The remaining eligible participants did not respond to the invitation to attend the online session and were therefore not randomized:

“A total of 103 participants responded to the invitation to attend the online session and were randomised…”.

- results: it is not clear what is meant with the following sentence '39 of whom were recruited within 2 months of active recruitment (rather than calendar time) following an amendment to gift inexpensive headsets, sent to participants via post.' Please consider rephrasing.

We have now clarified this on p.12:

“Due to the need for a protocol amendment, the study was first paused to await ethical approval and next paused for a couple of weeks when the researchers were on leave for the summer holidays. We therefore consider here only the active recruitment periods.”

**Reviewer #2**

This study sets out to examine the feasibility and acceptability of a pilot RCT using a brief virtual reality (VR) intervention in 60 smokers unmotivated to stop, randomized to an intervention (motivational VR) or control condition (control VR). Results showed that the targeted sample size was not achieved within the planned time frame, but that the intervention was perceived as acceptable. Preliminary analysis of the intervention effect on motivational constructs showed comparable point estimates in perceived susceptibility, response-efficacy and self-efficacy, and behavioral intention.

Overall, this paper takes an interesting approach using VR to deliver motivational messages. The manuscript is well structured and written and the experimental design is a strength. I however also have several concerns that narrow the impact of the manuscript. Overall, I find that in several instances the paper is rather short on information and appears superficial. I am also struggling that the sample recruitment is treated as the primary outcome and substantive questions which most likely are more relevant for the broader scientific community, are moved into the background. Also, there are several methodological concerns concerning the validity of measures and analysis (e.g., dichotomization). Please see more specific comments below:

We thank the reviewer for their helpful comments on our paper, which have helped strengthen it. We have now added detail in several places, as requested. As this was a pilot study with limited statistical power, we decided to focus on the feasibility of recruitment. Although we agree the other research questions are very interesting, we were not powered to substantively address these.

Introduction:

- Please elaborate on EPPM and what the theory-basis for the intervention scenario is.

We have now provided further detail about the theoretical underpinning of the brief VR scenario on p.3-4:

“We therefore helped develop an immersive, brief VR scenario – suited for delivery in smokers’ own homes – grounded in the Extended Parallel Process Model (EPPM) (Witte, 1992). The EPPM proposes that the effect of threatening health messages (e.g., information or communications highlighting that smoking causes lung cancer) on people’s motivation to protect themselves against said threat (e.g., through behaviour change) depends on two things: 1) people’s emotional responses to the message, and 2) their response- and self-efficacy beliefs (i.e., beliefs that the suggested action to avoid the threat is effective and that one has the ability to act). Previous research guided by the EPPM involving pictorial health warning labels on cigarette packages has found an association of greater self-efficacy and stronger emotional responses to health warning labels with prospective quit attempts (Thrasher et al., 2016). We therefore developed a hospital-based VR scenario, in which participants are invited to attend a consultation with a chest physician in a hospital clinic room and are told that they have had an abnormal chest scan which requires follow-up (i.e., a threatening health message). They are subsequently presented with a brief, written message to boost their response- and quitting self-efficacy. The hypothesised mechanism of action of the EPPM-based scenario is increased susceptibility to smoking-related diseases (i.e., a health threat) combined with increased response- and quitting self-efficacy, which are jointly expected to increase participants’ intention to stop smoking.”

- It is unclear if authors are interested in the acceptability of VR per se or the specific content delivered via VR in the study.

We have clarified on p.6 that we were interested in the acceptability of the specific intervention scenario delivered via VR. Our control was also delivered in VR – hence, there were no 2D controls to examine the comparative acceptability of VR per se:

“As both the intervention and control scenarios were delivered in VR, we aimed to assess the acceptability of the specific content delivered in VR format (i.e., there were no 2D controls to examine the comparative acceptability of VR per se).”

- Please elaborate on the concept of acceptability of digital health interventions as mutlifaceted concept and provide a rationale for why this is operationalized as attitudes.

The concept and operationalisation of acceptability have been clarified further on p.6:

“First, acceptability was examined by assessing participants’ affective and cognitive attitudes towards the respective scenarios (Sekhon et al., 2017). As both the intervention and control scenarios were delivered in VR, we aimed to assess the acceptability of the specific content delivered in VR format (i.e., there were no 2D controls to examine the comparative acceptability of VR per se). Although there is no consensus definition of acceptability, we recently proposed that acceptability may usefully be considered an emergent property of a complex, adaptive system of multiple, interacting components (e.g., beliefs, knowledge), which is experienced by the individual as a gut reaction or sudden insight (Perski & Short, 2021). Until further questionnaire development and validation works has been conducted, we reasoned that this sudden insight may be usefully operationalised as affective and cognitive attitudes.”

Methods:

- Please provide citations for where the items stem from and provide reliability coeffients along with descriptive data.

We have clarified that the affective and cognitive attitude items were developed for the purposes of the present study on p.7.

“These items were developed for the purposes of the present study and have not been validated.”

We have added to the limitations section on p.15 that the items used to capture acceptability were not validated.

“Finally, the items used to capture acceptability and intention to stop smoking were developed for the purposes of the present study and had not been validated.”

- Why were all measures dichotomized and on what basis? This is in my view highly problematic, resulting in a loss of information, and endangering validity.

The decision to dichotomise all measures was made to aid interpretation. We now present the results without dichotomisation in the Supplementary Materials 3 and refer to the results from these sensitivity analyses in the Results section.

“As a result of the peer review process, we also present the results without dichotomisation in the Supplementary Materials 3.”

“The results remained similar in the sensitivity analysis without dichotomisation (see the Supplementary Materials 3).”

- How valid is the behavioral measure of intention? I am not convinced that clicking on additional evidence-based information reflects intention to stop. It is also plausible that smokers who have
been motivated might not think they need further information to make a decision.

We are not aware of prior studies validating the behavioural indicator of intention to stop but have ourselves previously used a similar indicator in a different behaviour change context (<https://journals.plos.org/plosone/article?id=10.1371/journal.pone.0259949>). Prior research has shown that intention to change is positively associated with information seeking and user engagement in digital health (https://journals.sagepub.com/doi/pdf/10.1177/1359105308095064). We now discuss the lack of validation in the limitations section on p.15. From our knowledge of unmotivated smokers (who seek support at lower rates than those who are motivated), we judge it implausible that they would have felt adequately motivated to stop and thought that they didn’t need further information.

“Sixth, the items used to capture acceptability and intention to stop smoking were developed for the purposes of the present study and had not been validated. Though, we have previously used a similar behavioural indicator of intention to change in a different behaviour change context (Perski et al., 2021) and we note that prior research has found a positive association between intention to change and information seeking/user engagement in digital health research (Crutzen et al., 2008). Future research would benefit from assessing the prospective validity of such behavioural indicators of intention (i.e., whether website clicks are associated with self-reported smoking cessation attempts measured at a later time point).”

- Please elaborate the presented rationale for using point estimates (%). Why was it not possible to conduct regression analyses and report mean level differences and and corresponding CI (instead of P values)?

Due to the small sample size, we opted for a more descriptive approach (as already stated on p.10). In addition, when looking at the data following our decision to use a more descriptive approach, it also became apparent that logistic regression models would not converge for some research questions (e.g., intention to stop), as these require at least a few instances of each binary class (and the event rate in the control group was 0).

Discussion:

- There is no discussion of Research Question 3

We have now added the following on p.13:

”The perceived susceptibility and response-efficacy to cancer, heart disease, and lung disease were comparably high across arms, which may be indicative of a ceiling effect (i.e., participants already felt moderately to highly susceptible). Quitting self-efficacy and intention to stop smoking were comparably low across arms. There are different plausible interpretations of this finding. First, the brief VR scenario may have been insufficiently immersive or persuasive for boosting smokers’ self-efficacy and therefore also their motivation to stop. Second, smokers may have slightly rushed through the post-task survey and hence missed the link with more information about smoking cessation. This merits further exploration in, for example, a study using think aloud methodology, with smokers verbalising their thoughts and impressions whilst viewing the VR scenario.”

Minor points:

p. 5: explain why cancer patients were not eligible to take part in the study

We have clarified on p.5 that:

“Given the nature of the hospital-based VR scenario, which aimed to increase smokers’ perceived susceptibility to cancer (described in detail in section 2.5 below), we did not judge it ethical to include participants with a cancer diagnosis. Therefore, these participants were not eligible to take part, assessed by asking: “Have you been diagnosed with cancer?”.

p. 11: What is meant by calendar time?

We have now clarified this section in response to a comment from Reviewer #1:

“Due to the need for a protocol amendment, the study was first paused to await ethical approval and next paused for a couple of weeks when the researchers were on leave for the summer holidays. We therefore consider here only the active recruitment periods.”

**Reviewer #3**

This paper provides a clear report of the pilot RCT conducted by the authors. I have a number of minor comments that could be considered before the article is suitable for publication.

We thank the reviewer for their helpful feedback on our paper, which has helped improve it.

p. 3: suggest specifying what exactly is meant by an immersive VR scenario at this stage in the article (i.e., what sensory substitutions are necessary to facilitate this experience – vision, hearing, touch – and what equipment is absolutely necessary for the user to access the intervention). A more technical definition of immersive VR in the context of this study would also be more informative, offering a greater understanding of what the end-user is experiencing.

We have now provided a definition of immersive VR on p.3-4 and further detail about the current VR scenario (equipment needed, senses engaged):

“Although feelings of immersion (e.g., transportation to a different, virtual environment, losing track of time) are characteristic of VR more broadly, the level of immersion experienced by users differs depending on the specific technology used, the number of senses engaged (e.g., vision, hearing, smell) and the level of interactivity (e.g., whether users can interact with objects in the virtual environment through, for example, a joystick) (Miller and Bugnariu, 2016).”

“…suited for delivery in smokers’ own homes and accessible via commercially available head-mounted displays without involving a joystick…”

“With regards to the level of immersion, users are not able to interact with the physician via, for example, a joystick and the primary senses engaged are vision and hearing (but not smell, taste or haptics).”

p. 3: further description of the specific features (presence/co-presence, immersion) of immersive VR that are being utilized in this intervention would be important here due to the novelty of the technology – particularly in this field. Linked to this, as immersion appears to be a key hypothesised determinant of research question 3, further discussion about immersion as a phenomenon would strengthen the rationale for exploring the use of immersive VR in this context.

We have now amended the paragraph on p.3-4 to clarify the level of immersion in the current VR scenario (and how the scenario was expected to lead to increased self-efficacy and motivation to stop):

“We therefore helped develop an immersive, brief VR scenario – suited for delivery in smokers’ own homes and accessible via commercially available head-mounted displays without involving a joystick – grounded in the Extended Parallel Process Model (EPPM) (Witte, 1992). The EPPM proposes that the effect of threatening health messages (e.g., information or communications highlighting that smoking causes lung cancer) on people’s motivation to protect themselves against said threat (e.g., through behaviour change) depends on two things: 1) people’s emotional responses to the message, and 2) their response- and self-efficacy beliefs (i.e., beliefs that the suggested action to avoid the threat is effective and that one has the ability to act). Previous research guided by the EPPM involving pictorial health warning labels on cigarette packages has found an association of greater self-efficacy and stronger emotional responses to health warning labels with prospective quit attempts (Thrasher et al., 2016). We therefore developed a hospital-based VR scenario, in which participants are invited to attend a consultation with a chest physician in a hospital clinic room and are told that they have had an abnormal chest scan which requires follow-up (i.e., a threatening health message). They are subsequently presented with a brief, written message to boost their response- and quitting self-efficacy. The hypothesised mechanism of action of the EPPM-based scenario is increased susceptibility to smoking-related diseases (i.e., a health threat) combined with increased response- and quitting self-efficacy, which are jointly expected to increase participants’ intention to stop smoking. With regards to the level of immersion, users are not able to interact with the physician via, for example, a joystick and the primary senses engaged are vision and hearing (but not smell, taste or haptics).”

p. 8: suggest including any data available on the user testing conducted in December 2020 (researcher notes/observations, reflexive notes, etc.) as it would offer more insight into the processes involved in designing this intervention as suggested in the recommendations offered at the end of this article – further qualitative research into end-users’ experiences and the application of user-centred design principles.

The user testing was pragmatic in nature and focused on making the entire study flow easy for participants to understand. We therefore do not have additional insights to share about end-users’ experiences (apart from those presented) and have already highlighted this as an important avenue for future research in the Discussion section.

p. 12: if the data is available, it would be useful to know what other immersive VR devices participants who already had the equipment were using. Current immersive VR technologies can differ significantly, affecting key experiences such as presence and immersion.

We have now presented this information in Table 1.

p. 13: suggest supporting claims of novelty effects and the effects varying levels of immersion have on immersive VR participants’ experiences with past research.

We have now added references in support of a potential novelty effect (<https://pubmed.ncbi.nlm.nih.gov/33296309/>; <https://ieeexplore.ieee.org/document/9388893>) in addition to the potential differential effectiveness of VR scenarios with varying levels of immersion on psychological outcomes (https://www.ncbi.nlm.nih.gov/pmc/articles/PMC4827274/) on p.14.

p. 13: typological error – “third” used twice when outlining limitations

Thank you – this has now been amended.
