# Supplementary material for: A pilot randomised trial of a brief virtual reality scenario in smokers unmotivated to quit: Assessing the feasibility of recruitment
Source: PLOS Digit Health. 2022 Jun 27;1(6):e0000060. doi: 10.1371/journal.pdig.0000060 (PMC9931367; doi:10.1371/journal.pdig.0000060)
Supplement: Attachment — Submitted filename: Responses to reviewer comments 01.05.22.docx [file pdig.0000060.s005.docx]

**Editor**

Based on additional comments submitted by one of the reviewers, I would like to ask the authors to make the following changes:

We thank the Editor for their helpful comments on our manuscript and for the opportunity to revise it.

- Briefly summarize the results presented in the supplementary material in the main text to contextualize the meaning of "remained similar".

In response to the reviewer comments, we have now updated the sensitivity analyses and summarise the findings on p.12:

“The intervention (86.7%, 95% CI=69.3%-96.2%) and control (93.3%, 95% CI=77.9%-99.2%) scenarios were rated as acceptable. In the sensitivity analysis with the acceptability indicators operationalised as continuous variables, there were no significant differences between the intervention and control scenarios (see the Supplementary Materials 3).”

“Proximal quitting outcomes in the intervention and control arm were comparable (see Table 2). In the sensitivity analysis without the proximal quitting outcomes operationalised as continuous variables (except for intention to stop), there were no significant differences between the intervention and control scenarios, with the exception of quitting self-efficacy (see the Supplementary Materials 3). Quitting self-efficacy was significantly greater in the control (M = 3.03, SD = 1.07) compared with the intervention arm (M = 2.30, SD = 0.95), p = 0.006.”

- The authors state that the concept of acceptability is debated; this debate should be reflected in the discussion.

We now emphasise this debate in the Discussion on p.14:

“With regards to the definition and operationalisation of intervention acceptability, this remains a debated topic and we have previously argued that acceptability can usefully be conceptualised as an emergent property of a complex, adaptive system of interacting subcomponents (e.g., cognitive and affective attitude) (Perski & Short, 2021). However, it should be noted that there may be some degree of overlap between acceptability as operationalised in the present study (e.g., anger, distress, perceived usefulness) and the emotional reactions expected to be elicited by the VR scenario (e.g., fear, worry) – which in turn were expected to influence perceived susceptibility to cancer/heart disease/lung disease. Although correlations between the acceptability and susceptibility indicators were small in magnitude and non-significant (presented in the Supplementary Materials 3), further conceptual and psychometric work is required to refine the acceptability measure and evaluate its validity and reliability (including the identification of a suitable cut-off score across the three indicators).”

- The reviewer highlights that acceptability and susceptibility might be strongly correlated due to the wording of the items. This is a valid concern should be addressed.

Please see our response to the previous comment.

**Reviewer #2:**

I would like to thank the authors for taking the time and effort to revise their manuscript. I appreciate the clarifications throughout the manuscript, but still have a few questions or concerns, mainly regarding to the operationalization and validity of measures:

We thank the reviewer for their helpful comments on our manuscript, which have helped strengthen it.

1. I am not fully convinced that the benefit of the dichotomization in aiding the interpretation of results outweighs the concern of loss of information. The concepts measured clearly reflect continuous concepts and a clear rationale for the cutoffs chosen is missing. From my personal viewpoint, the previously planned analyses with regressions (maybe except for intention to change) would yield more informative results.

We now present sensitivity analyses (including a series of linear regression analyses) in the Supplementary Materials 3 with the outcome variables (except for intention to stop) operationalised as continuous variables. There were no significant differences between the intervention and control arms, except for quitting self-efficacy. We summarise these findings on p.12:

“The intervention (86.7%, 95% CI=69.3%-96.2%) and control (93.3%, 95% CI=77.9%-99.2%) scenarios were rated as acceptable. In the sensitivity analysis with the acceptability indicators operationalised as continuous variables, there were no significant differences between the intervention and control scenarios (see the Supplementary Materials 3).”

“Proximal quitting outcomes in the intervention and control arm were comparable (see Table 2). In the sensitivity analysis without the proximal quitting outcomes operationalised as continuous variables (except for intention to stop), there were no significant differences between the intervention and control scenarios, with the exception of quitting self-efficacy (see the Supplementary Materials 3). Quitting self-efficacy was significantly greater in the control (M = 3.03, SD = 1.07) compared with the intervention arm (M = 2.30, SD = 0.95), p = 0.006.”

2. As to the additional analyses in Supplementary File 3, can the authors explain what “remain similar” means? Again, I belief that reporting (descriptive) means intervention vs. control would be more informative than proportions across the likert scale.

Please see our response to the previous comment.

3. I am still struggling to grasp as to how the affective beliefs should capture acceptability. One item example is “made me feel distressed”, and if participants report that the disagreed this was coded as acceptable. However, the scenario was expected to raise emotions, so distress might reflect that health consequences were perceived as intended in the scenario (similar to a manipulation check)? This might pose a problem for the measure of acceptability. Can the authors please explain how this should conceptualize non-acceptability? Also, was a mean score of cognitive and affective attitudes calculated? If yes, please explicitly state so.

We appreciate this concern and although we argue that two out of three acceptability indicators (anger, perceived usefulness) are clearly delineated from the emotions expected to be linked to perceived susceptibility to cancer/heart disease/lung disease (fear, worry), distress could arguably be linked to either. However, as acceptability is conceptualised as an emergent property of a complex, adaptive system of interacting subcomponents, we opted to generate a combined measure of acceptability in the present study (i.e., not relying on single measures of anger, distress, or perceived usefulness). In response to this and the next comment, we have calculated bivariate correlations between the acceptability and susceptibility indicators, which were all small in magnitude and non-significant. We do, nonetheless, agree that further conceptual and psychometric work is required to refine measures of intervention acceptability (in addition to the identification of suitable cut-offs for acceptability/non-acceptability), and now emphasise this in the Discussion on p.14:

“Sixth, the items used to capture acceptability and intention to stop smoking were developed for the purposes of the present study and had not been validated. Though, we have previously used a similar behavioural indicator of intention to change in a different behaviour change context (Perski et al., 2021) and we note that prior research has found a positive association between intention to change and information seeking/user engagement in digital health research (Crutzen et al., 2008). With regards to the definition and operationalisation of intervention acceptability, this remains a debated topic and we have previously argued that acceptability can usefully be conceptualised as an emergent property of a complex, adaptive system of interacting subcomponents (e.g., cognitive and affective attitude) (Perski & Short, 2021). However, it should be noted that there may be some degree of overlap between acceptability as operationalised in the present study (e.g., anger, distress, perceived usefulness) and the emotional reactions expected to be elicited by the VR scenario (e.g., fear, worry) – which in turn were expected to influence perceived susceptibility to cancer/heart disease/lung disease. Although correlations between the acceptability and susceptibility indicators were small in magnitude and non-significant in the present study (presented in the Supplementary Materials 3), further conceptual and psychometric work is required to refine the acceptability measure and evaluate its validity and reliability (including the identification of a suitable cut-off score across the three indicators). In addition, future research would benefit from assessing the prospective validity of behavioural indicators of intention (i.e., whether website clicks are associated with self-reported smoking cessation attempts measured at a later time point).”

4. What is the correlation among the variables assessed. As with regard to my second concern, I am wondering if acceptability might correlate/overlap strongly with the measure of susceptibility.

Please see our response to the previous comment.
